# CERES–Maize Model for Determining the Optimum Planting Dates of Early Maturing Maize Varieties in Northern Nigeria

**DOI:** 10.3389/fpls.2017.01118

**Published:** 2017-06-28

**Authors:** Adnan A. Adnan, Jibrin M. Jibrin, Alpha Y. Kamara, Bassam L. Abdulrahman, Abdulwahab S. Shaibu, Ismail I. Garba

**Affiliations:** ^1^Department of Agronomy, Centre for Dryland Agriculture, Bayero University KanoKano, Nigeria; ^2^Department of Soil Science, Centre for Dryland Agriculture, Bayero University KanoKano, Nigeria; ^3^International Institute for Tropical AgricultureIbadan, Nigeria

**Keywords:** CERES–maize, planting date, early maize, sensitivity analysis, Northern Nigeria

## Abstract

Field trials were carried out in the Sudan Savannah of Nigeria to assess the usefulness of CERES–maize crop model as a decision support tool for optimizing maize production through manipulation of plant dates. The calibration experiments comprised of 20 maize varieties planted during the dry and rainy seasons of 2014 and 2015 at Bayero University Kano and Audu Bako College of Agriculture Dambatta. The trials for model evaluation were conducted in 16 different farmer fields across the Sudan (Bunkure and Garun—Mallam) and Northern Guinea (Tudun-Wada and Lere) Savannas using two of the calibrated varieties under four different sowing dates. The model accurately predicted grain yield, harvest index, and biomass of both varieties with low RMSE-values (below 5% of mean), high d-index (above 0.8), and high *r*-square (above 0.9) for the calibration trials. The time series data (tops weight, stem and leaf dry weights) were also predicted with high accuracy (% RMSEn above 70%, d-index above 0.88). Similar results were also observed for the evaluation trials, where all variables were simulated with high accuracies. Estimation efficiencies (EF)-values above 0.8 were observed for all the evaluation parameters. Seasonal and sensitivity analyses on Typic Plinthiustalfs and Plinthic Kanhaplustults in the Sudan and Northern Guinea Savannas were conducted. Results showed that planting extra early maize varieties in late July and early maize in mid-June leads to production of highest grain yields in the Sudan Savanna. In the Northern Guinea Savanna planting extra-early maize in mid-July and early maize in late July produced the highest grain yields. Delaying planting in both Agro-ecologies until mid-August leads to lower yields. Delaying planting to mid-August led to grain yield reduction of 39.2% for extra early maize and 74.4% for early maize in the Sudan Savanna. In the Northern Guinea Savanna however, delaying planting to mid-August resulted in yield reduction of 66.9 and 94.3% for extra-early and early maize, respectively.

## Introduction

The total annual national production of maize in Nigeria has increased from 0.66 M tons in 1978, to about 11.3 M tons in 2013 (FAOSTAT, FAO, 2014). Despite the increased area under maize production, yields have remained quite low. The average yield of maize in Nigeria was 1.4 tons ha^−1^ in 2013 compared to 9.5 tons ha^−1^ in the USA and the world average of 5.5 tons ha^−1^ (FAOSTAT, FAO, 2013). The major factors limiting the yield of maize in Nigeria include the inherently poor soils (Jibrin et al., 2012), frequent droughts (Kamara et al., 2009), lack of proper adherence to improved agronomic practices (especially planting dates and densities) and low use of improved inputs such as fertilizers and seeds (Badu-Apraku et al., 2009). Maize production in Nigeria was initially restricted to the Derived Savanna and Humid Forests due to high amounts of annual precipitation (Sowunmi and Akintola, 2010). In recent years, new early and extra early maturing maize varieties have been developed for the wet and dry Savannas of Nigeria because of the short growing periods in these areas (Badu-Apraku et al., 2011). The Nigerian Savannas are divided into Guinea Savanna and Sudan Savannah. This classification is based on the similarity of climatic elements and the type of vegetation that can be supported (Ogungbile et al., 1998; Sowunmi and Akintola, 2010).

It has been generally agreed that in order to increase maize production in the Nigerian Savannas, production practices should be properly designed to tolerate the low precipitation and high temperatures that characterize the zone (Jibrin et al., 2012). Growing adaptable maize cultivars and choosing optimum planting dates are avenues to increase yields that farmers can adopt. Because of the short growing season, early and extra-early maturing maize cultivars with drought tolerance are desired (Kamara et al., 2009). The optimum planting date for early maturing maize in the Sudan and Northern Guinea Savannas has been reported to be the last week of June, while extra-early maturing varieties are planted in first or second week of July (Jaliya et al., 2008; Kamara et al., 2009). In the Savannas of Nigeria, the length of growing season is determined by the date of first rains and thus is highly variable from year to year. Climate change (majorly rise in temperatures) has led to a shift in the onset of the rainy season. In most areas of West and Central Africa, delays in onset of the rainy season has been consistently observed (Graef and Haigis, 2001; Marteau et al., 2011). Also, long dry spells at the beginning, mid and end of the rainy season are becoming more frequent even in the wetter Southern and Northern Guinea Savannas. As a result of these constraints, rainfed agricultural production is becoming more variable, and farmers are faced with more risks during production, as a result, optimal timing of all production practices is becoming more important (Staggenborg et al., 1999). It becomes necessary for producers to know the extent to which planting can be delayed and also the likely yield penalty they could experience as a result of late planting.

Recommendations for planting dates of maize are usually based on agronomic field experiments that are specific to fields and regions (Sorensen et al., 2000). Majority of such trials cannot be temporally and spatially replicated because of seasonal variations. Determination of optimum sowing dates for maize by field experimentation entails repetition over long periods of time in order to capture seasonal variability in precipitation. Also, data for one location is not useful for another location because of variation in not only rainfall but edaphic factors as well. Decision support tools (DSTs) therefore remain very important diagnostic tools for analysis of options that relate to sowing date rules and other crop management strategies. DSTs such as crop simulation models are not widely used in sub-Saharan Africa due to lack of knowledge.

Simulation models have been developed as tools to support strategic decision-making in research, production, land use and policy (Penning de Vries et al., 1993). These models can be used to evaluate agricultural production risk as a function of climate variability, to assess regional yield potential across a wide range of environmental conditions and to determine fertilizer applications, suitable planting dates, and other management factors for increasing crop yield (Egli and Bruening, 1992; Hunt and Boote, 1998; Kaur and Handal, 1999). There are several different crop and soil simulation models available to simulate maize growth and management, such as agricultural production systems simulator (APSIM; Keating et al., 2003), a cropping systems simulator (CropSyst; Stöckle et al., 2003), erosion-productivity impact calculator (EPIC; Jones et al., 1991; Williams, 1995), and decision support system for agro-technology transfer (DSSAT; Jones et al., 2003). CERES–maize model is a module within the DSSAT cropping system model (CSM). The DSSAT CSM can facilitate the evaluation of the effects of different production practices on crop yields, growth rates, and nutrient losses, and also it helps improve our understandings of crop physiology, genetics, soil management, and weather effects on crop production and environmental quality (Cabrera et al., 2007; Boote et al., 2010). The DSSAT CSM uses common soil C/N and water models, which integrate mathematical equations to describe the transformation and fluxes of various components of the of soil carbon, water and nutrient cycles on a daily or hourly basis. At the same time, it also predicts the temporal changes in crop growth, nutrient uptake, water use, final yield as well as other plant traits, and outputs (Boote et al., 2010). Therefore, the dynamic CSM can integrate the effects of soil management and climate, which enable us to predict the impact on crop production and environmental quality.

CERES–maize model has been found to be able to accurately predict yield variability, N uptake and maize growth response to nitrogen (Pang et al., 1997; Bert et al., 2007) and to assess site-specific nitrogen management to maximize field level net return and minimize environmental impact by using spatially variable management practices (Paz et al., 1999; Batchelor et al., 2002; Link et al., 2006; Miao et al., 2006; Thorp et al., 2008). Gungula et al. (2003) employed CERES-maize to simulate maize phenology under nitrogen-stressed conditions in Nigeria, and showed that the model could be reliably used for predicting maize phenology only under non-limiting N conditions and then suggested that an N stress factor is required to predict crop phenology in low-N tropical soils. Jagtap et al. (1993) reported that CERES–maize model predicted grain yield, stalk and leaf weight, and aboveground biomass within 10% of the field observed data, which means that the built-in partitioning rules in the model are robust and adequate. Soler et al. (2008), used CERES–millet model to determine optimum planting dates of millet in Niger Republic. Wolf et al. (2015), used CERES–maize model to identify sowing rules for estimating rainfed yield potential of sorghum and maize in Burkina Faso.

The objective of this study is to calibrate and evaluate the CSM–CERES–maize model's ability in simulating yield of early and extra early maturing maize varieties in the Savannas of Nigeria and evaluate the ability of the model in simulating yield of maize under varying planting dates in contrasting environments.

## Materials and methods

### Field experiments

Two different sets of experiments were conducted for model calibration and evaluation. For model calibration, eight (8) field trials were conducted in the rainy and dry seasons of 2014 and 2015 at the Bayero University, Kano (BUK) Agricultural Research Farm (11°59′N; 8°25′E; 466 m asl) and Audu Bako College of Agriculture Dambatta (12°19′N; 8°31′E; 504 m asl). The dry-season experiments were conducted under irrigated conditions between early March to early June 2014 and 2015, while the rainy season experiments were planted under rainfed conditions with supplementary irrigations between mid-June to early October 2014 and 2015. The experiments were laid out as a single factor experiment in a randomized complete block design (RCBD) with three replications. Twenty maize varieties (only two were used in the present study due to popularity and utilization in the study areas) were randomized and assigned to plots, plot sizes were six ridges (0.75 m between ridges) each 5 m in length making each main plot 30 m^2^ [(8 × 0.75 m = 6 m) × 5 m]. Planting was done at a spacing of 25 cm between stands and 75 cm between rows, two seeds were planted and later thinned to one stand at 2 weeks after sowing. NPK fertilizers were applied according to soil analysis so as to ensure optimum nutrient availability. Detailed soil and weather information from each location and season were collected according to the minimum data sets required for calibration of CERES–maize model as suggested by Jones and Kiniry (1986). All data collections were done in the two inner rows, 50 cm from each end of the ridge were ignored and all plants inside were used as net plot, making the net plot size to be 6 m^2^. Profile pits were dug prior to the start of experimentation for soil characterization in both locations. A Time Domain Reflectometer (TDR, FieldScout TDR300, by Spectrum Technologies, Inc.) was used to measure soil moisture content throughout the period of experiment; and supplementary irrigation was given when readily available water (RAW) was fully depleted in order to ensure optimal moisture availability.

The second sets of experiments were conducted for the purpose of model evaluation. On-farm trials were set in 16 farmers' fields across the Sudan and Northern Guinea Savannas of Nigeria in 2014. The experiments were conducted in Bunkure, Garun-Mallam, Tudun-Wada, and Lere local governments. The evaluation trials were set under researcher managed conditions in farmers' field. The treatments for the evaluation trials includes one early (EVDTW2009STR) and one extra-early maize variety (2009TZEEWDTSTR) under four different planting windows (Early June, Mid-June, Early July, and Mid-July). Planting was done on 5th June, 16th June, 3rd July, and 17th July across all locations. Optimum fertilizer recommendations were used in all locations, two seeds were planted at an intra-row spacing of 0.25 m, and later thinned to one seedling per stand at 2 weeks after planting. The plots for evaluation trials were eight ridges (0.75 m apart) by 5 m length which gave a plant population of 53,333 plants ha^−1^. All recommended agronomic practices for the areas were strictly followed.

### Pedo-climatic conditions

Table 1 shows the soil properties of pedons in BUK and Dambatta experimental sites. Pedons 1 and 2 represents the 2014 and 2015 trials at BUK, while pedons 3 and 4 represents the 2014 and 2015 trials at Dambatta. The surface horizon at BUK experimental site had a Loamy sand texture, slightly acidic to neutral pH, low organic carbon content, and medium level of total nitrogen. The available phosphorus was in the medium fertility class while cation exchange capacity was low in both 2014 and 2015. On the other hand, the surface horizon at Dambatta experimental site had a sandy loam to loamy sand texture, moderately acidic to slightly acidic pH, low organic carbon content, and medium level of total nitrogen. The available phosphorus was in the medium fertility class while cation exchange capacity was also low in both 2014 and 2015. The pedons were classified according to the USDA Soil Taxonomy (Soil Survey Staff, 2014).

**Table 1:** Physical and chemical properties of Pedons of the 2014 and 2015 rainy and dry season calibration experiments at BUK and Dambatta.

**Profile**	**Horizon**	**Depth (cm)**	**Sand**	**Clay (g kg^−1^)**	**Silt**	**BD (g/cm^3^)**	**O.C (g kg^−1^)**	**pH (H_2_O)**	**pH (KCl)**	**%BS**	**EC (μs/m)**	**CEC (Cmol kg^−1^)**	**Av P (mg/kg)**	**T.N (g kg^−1^)**
**PEDONS OF THE 2014 AND 2015 RAINY AND DRY SEASON CALIBRATION EXPERIMENTS AT BUK**														
1	Ap	0–13	820	50	130	1.66	3.00	6.57	5.23	67.8	4.75	2.00	12.63	1.90
1	AB	13–47	800	70	130	1.72	1.80	6.77	5.89	67.8	6.05	1.10	5.76	1.50
1	BW1	47–74	860	50	90	1.60	0.80	6.99	4.98	67.8	4.25	2.20	4.53	1.50
1	BW2	74–97	860	50	90	1.67	0.60	7.15	5.76	89.9	3.20	4.40	3.98	1.10
1	BW3	97–130	860	70	70	1.70	0.60	7.17	5.89	60.6	3.50	1.80	5.42	1.20
1	BW4	130–184	840	90	70	1.70	0.40	7.28	4.78	88.6	3.70	2.20	3.65	1.10
2	Ap	0–15	830	120	50	1.56	2.40	6.81	4.92	52.4	5.85	3.5	13.95	1.80
2	AB	15–30	820	50	130	1.67	1.80	6.64	5.12	57.6	8.30	1.7	7.14	1.80
2	BW1	30–72	800	90	110	1.66	1.40	5.98	4.67	64.0	5.85	2.7	3.77	1.50
2	BW2	72–102	760	90	150	1.65	1.10	6.17	5.87	86.3	3.60	2.1	4.05	1.50
2	Bt	102–113	780	90	130	1.68	1.10	6.57	5.34	88.6	4.55	3.40	3.64	1.20
**PEDONS OF THE 2014 AND 2015 RAINY AND DRY SEASON CALIBRATION EXPERIMENTS AT DAMBATTA**														
3	Ap	0–10	780	90	130	1.34	8.50	5.87	5.23	67.8	4.75	2.00	8.75	1.80
3	AC	10–35	720	120	160	1.60	4.60	5.90	5.89	67.8	6.05	1.10	1.75	2.80
3	C1	35–60	820	60	120	1.67	0.40	6.40	4.98	67.8	4.25	2.20	1.75	1.10
3	C2	60–114	820	80	90	1.47	5.30	6.30	5.76	89.9	3.20	4.40	4.38	1.80
4	Ap	0–26	860	30	110	1.48	2.50	6.10	4.92	52.4	5.85	2.5	1.75	1.50
4	BA	26–63	840	30	130	1.31	2.10	5.90	5.12	57.6	8.30	4.1	1.75	1.80
4	BC	63–143	830	10	160	1.31‘	1.10	6.10	4.67	64.0	5.85	2.8	2.63	2.10
4	C	143–232	850	10	140	1.43	0.80	6.10	5.87	86.3	3.60	3.2	1.75	1.80

The soils for the evaluation sites were all similar to the calibration experiments. The Ap horizon for the soils in Bunkure had neutral pH of 6.6, organic carbon contents of 2.7 g/kg, available P of 12.72 mg/kg, and total nitrogen of 1.78 g/kg. In Garun-Mallam, the soils had slightly higher pH (6.62), more organic carbon contents (3.0), higher available P (13.0), and more total nitrogen 1.81 g/kg. In the northern Guinea Savanna, the soils from both locations had neutral pH, higher organic carbon contents (3.3 and 3.8 g/kg), higher available P-values (13.1 and 13.3 g/kg), and more total nitrogen (1.87 and 1.92).

**Figure 1:**
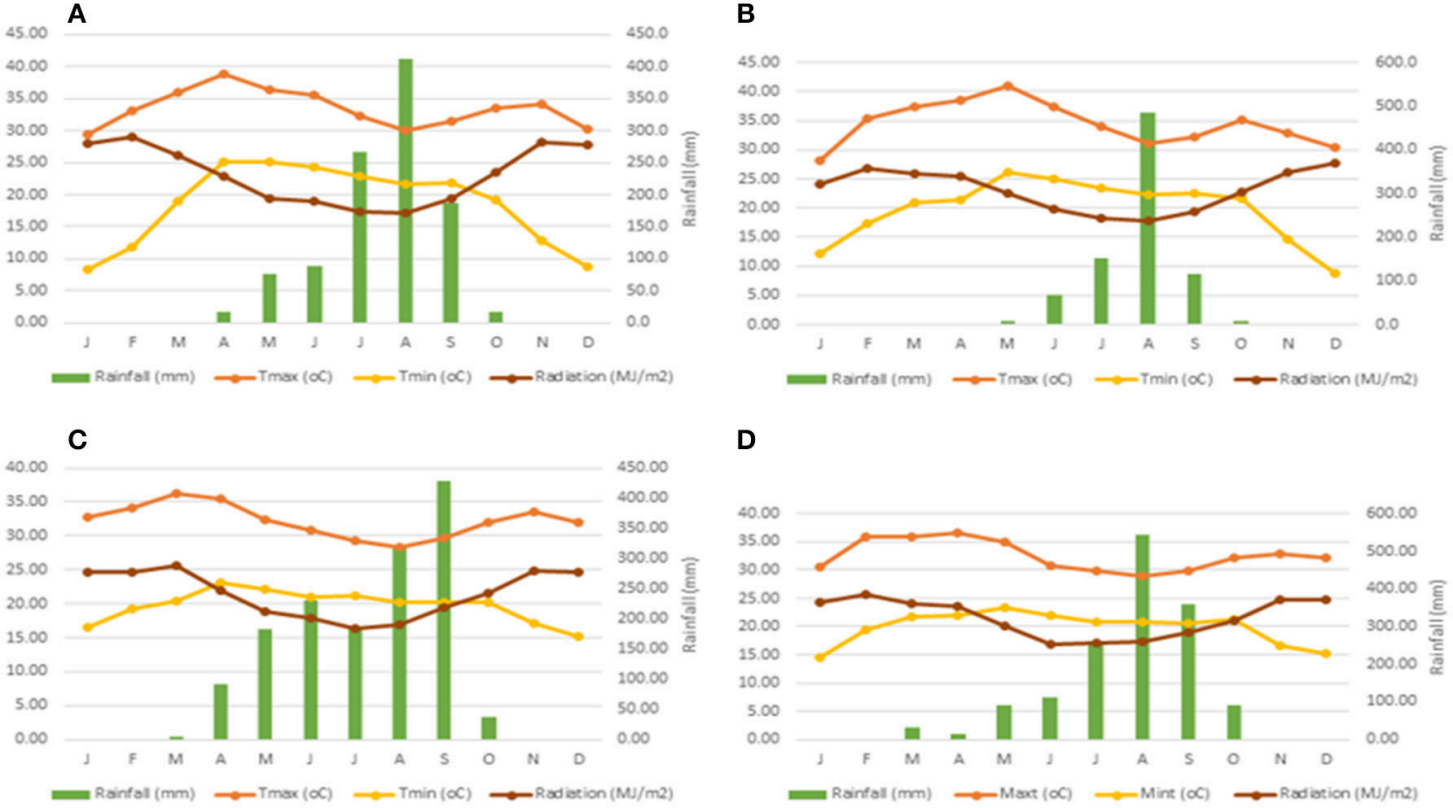
Total rainfall, mean, minimum, and maximum temperature and mean solar radiation for calibration sites: **(A)** BUK 2014; **(B)** Dambatta 2014; **(C)** BUK 2015; **(D)** BUK 2015.

**Figure 2:**
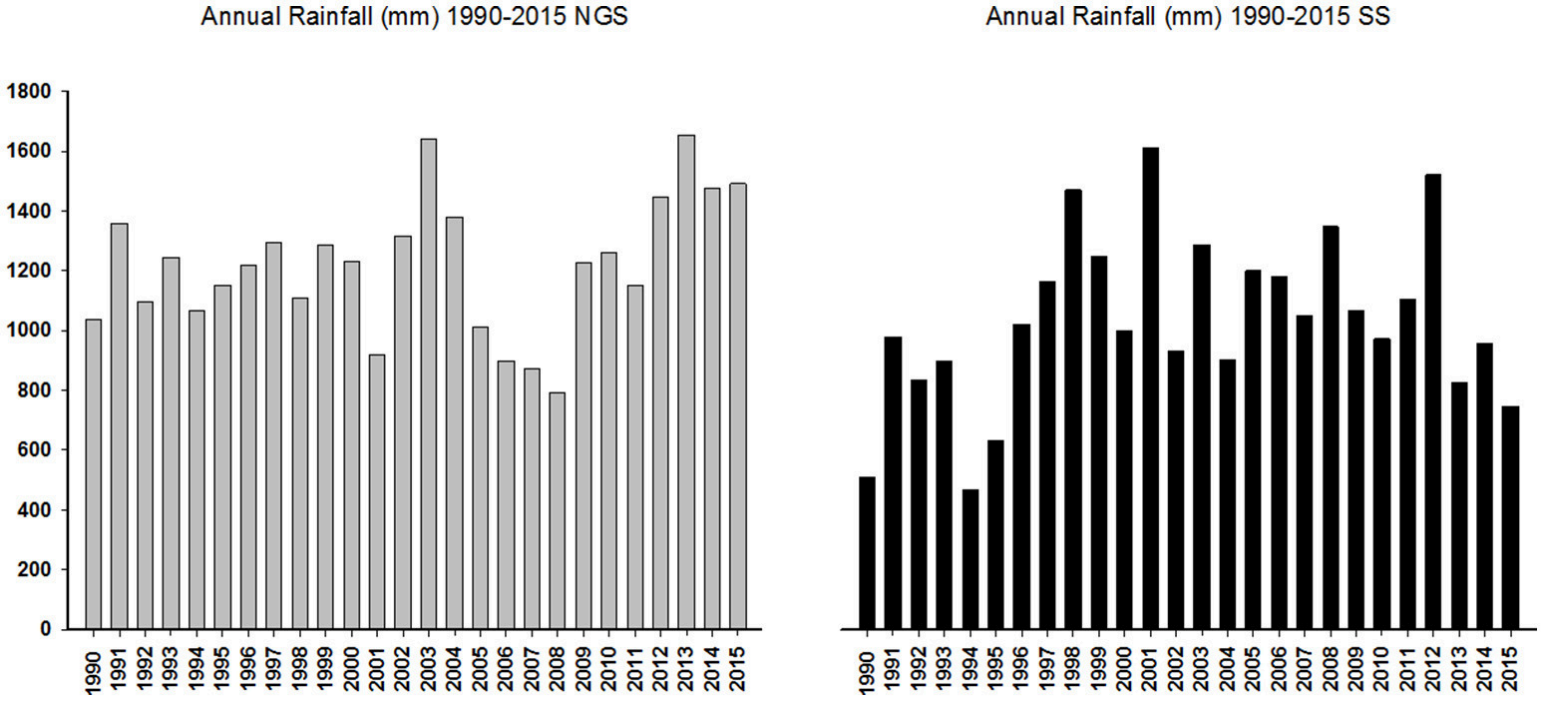
Twenty six years (1990–2015) total annual rainfall for Northern Guinea (Zaria) and Sudan Savanna (Bunkure).

Weather data for both years and experimental locations are shown in Figure 1. For the calibration experiments, weather data were collected from weather stations (Watchdog 2000 Series, Spectrum Technologies) adjacent to both experimental sites. For evaluation and sensitivity analysis however, weather data were obtained from the IITA Station in Kano and the Nigerian Meteorological Agency (NIMET). Higher amounts of rainfall were recorded in the NGS for both years as expected, while higher amount of rainfall was recorded in 2014 than in 2015. Figure 2 shows 26 years (1990–2015) total annual rainfall for Sudan Savanna (Bunkure) and Northern Guinea Savanna (Zaria).

### Plant measurements

Plant measurements used for model calibration were: grain yield at maturity, tops weight at anthesis, tops weight at maturity, and harvest index. While for model evaluation, grain yield at harvest, tops weight at harvest, and stalk weight at anthesis were measured. Phenological studies during vegetative stage were conducted by counting the leaves' collar appearance daily for each variety to be used in calibration. Tasseling and silking were recorded when tassels and silks become visible outside on 50% of the plants of each plot. For measurement of physiological maturity, regular sampling of two cobs per plot was done to assess the presence of black layers at the base of the grains. Destructive method of sampling was used to obtain above ground biomass by sampling 1 m of row from the sampling rows of each plots every 18 days. Sampled plants were separated into different parts and oven dried to constant weight and the weight recorded. Manual harvesting was done to determine the final harvest measurements. Harvest was conducted in the two middle ridges by harvesting 5 m × two ridges, with the ridges measuring 0.75 cm apart. Sampled plants were then separated into leaf, stem, ears, and husks and later oven dried before weighing. Kernel moisture was determined by collecting samples, weighing them, drying in an oven, and weighted again. Five plants were sampled per plot (15 per replication) to determine the average number of grains.

### CSM–CERES–maize model evaluation

#### Model calibration

The eight experiments (four in two locations) conducted in 2014 and 2015 were used for model calibration. The DSSAT model inputs include cultivar coefficients, weather data (min. and max. temperature, rainfall, and relative humidity), initial soil moisture, soil organic C, N and soil inorganic N and P, soil topography/surface information, such as slope, soil color, and crop management details (Jones et al., 1994). The major physiological processes (photosynthesis, respiration, accumulation, and partitioning of assimilates) in the CERES–maize model are governed by six genetic coefficients (**Table 3**) found in the maize cultivar file (Hoogenboom et al., 2010). The six parameters are user adjustable and they determine growth, phenology, and yield of the cultivars. For the purpose of this calibration, the sequential approach was adopted. Growing degree days (GDD) or thermal time, drive the phenological phase of development in the CERES–maize models. GDD is computed based on the daily maximum and minimum temperature (Equation 1). In some growth stages, day length is also considered (Jones and Kiniry, 1986; Jones et al., 2003).

(1) $$GDD=\frac{Tmax\;+Tmin}{2}-Tbase$$

Where GDD is growing degree days, *T*_max_ is maximum temperature, *T*_min_ is minimum temperature and *T*_base_ is base temperature (*T*_base_ for maize = 8°C). GDD is cumulative and is measured in °C day^−1^.

From the calibration experiments; P1, P5, G2, G3, and PHINT were estimated. In addition to the cultivar coefficients, two genetic coefficients [the soil fertility coefficient (SLPF), and the radiation use efficiency (RUE)] were also calibrated in order to be able to properly simulate above ground biomass and grain yield across locations and seasons. The *SLPF* was calibrated to optimize the soil variability across fields while the *RUE* optimized the variation across seasons. The Genotype Coefficient Calculator (GENCALC) of DSSAT 4.6 was used to estimate the maize cultivar coefficients. The statistics used for model calibration were r-square and RMSE, in addition normalized RMSE (RMSEn) was used for multiple targets because it is difficult to use RMSE alone (Anothai et al., 2008). RMSEn is shown in Equation (2), and it gives a normalized value that allows averaging over multiple characteristic targets providing a single index for their goodness of fit.

(2) $$RMSE=\sqrt{\frac{\sum_{i\;=\;1}^{n}\left(m_{i}-s_{i\;}\right)}{n}}$$

(3) $$RMSEn=\;\frac{RMSE\;\times 100}{\overset{-}{m}}$$

#### Model evaluation

The 16 on-farm experiments were used for model evaluation. Experiments were set in 16 locations across the Sudan and the Northern Guinea Savannas of Nigeria. The evaluation experiments were used to test the optimized parameters achieved from calibration experiments. The data used for evaluation were: days to anthesis, days to physiological maturity, grain yield at harvest, stalk weight at anthesis, and tops weight at harvest. Evaluation of model performance was done by calculating root mean square error (*RMSE*), model forecasting efficiency (*EF*), and mean error (*E*) based on previous model evaluation studies (Yang and Huffman, 2004). In addition, an index of agreement (*d*) statistic was employed in this study. The *d* statistic is recommended for making cross-comparisons when the *d*-value is both relative and has bounded measures (Willmott, 1982).

(4) $$EF=\;\frac{\sum_{i\;=\;1}^{n}{\left(m_{1}-\overset{-}{m}\right)}^{2}-\sum_{i\;=\;1}^{n}s_{i}-m_{i}}{\sum_{i\;=\;1}^{n}{\left(m_{i}-\overset{-}{m}\right)}^{2}}$$

(5) $$d=1-\;\frac{\sum_{i\;=\;1}^{n}{\left(m_{i}-S_{i}\right)}^{2}}{\sum_{i\;=\;1}^{n}|S_{i}|+|m_{i}|{)}^{2}}$$

Where is the number of measured dataset, *S*_*i*_ is the simulated data, *m*_*i*_ is the measured data, and $\overset{-}{m}$ is the mean of the measured data, $S_{i^{\prime }}=\;S_{i}-\overset{-}{m}$ and $m_{i^{\prime }}$ = $m_{i}-\overset{-}{m}$.

#### Sensitivity analysis (model application)

Sensitivity analysis was carried out to test the effect of varying planting dates on yield of maize in two locations; Bunkure in the Sudan Savanna and Zaria in the Northern Guinea Savanna. Generally, Bunkure had a shorter growing season with mean rainfall of 825 mm and growing season of 3.5 months. Average rainfall in the Zaria is 1,125 mm with growing period of 5 months. Historical weather records (1990–2015) were obtained from NIMET and used for seasonal analysis. Ten planting dates were simulated using the seasonal analysis tool of DSSAT 4.6. The planting dates started from 20th May and were repeated every 10 days until 20th August. Cumulative frequency plots were used to present the results of simulated yields over 26 years. Stable means for 26 years for each sowing date, variety and location were calculated together with maximum and minimum obtainable. In addition, percentage yield reduction for each planting date, locations, and varieties were calculated.

## Results

### CSM–CERES–maize model evaluation

#### Model calibration

**Figure 3:**
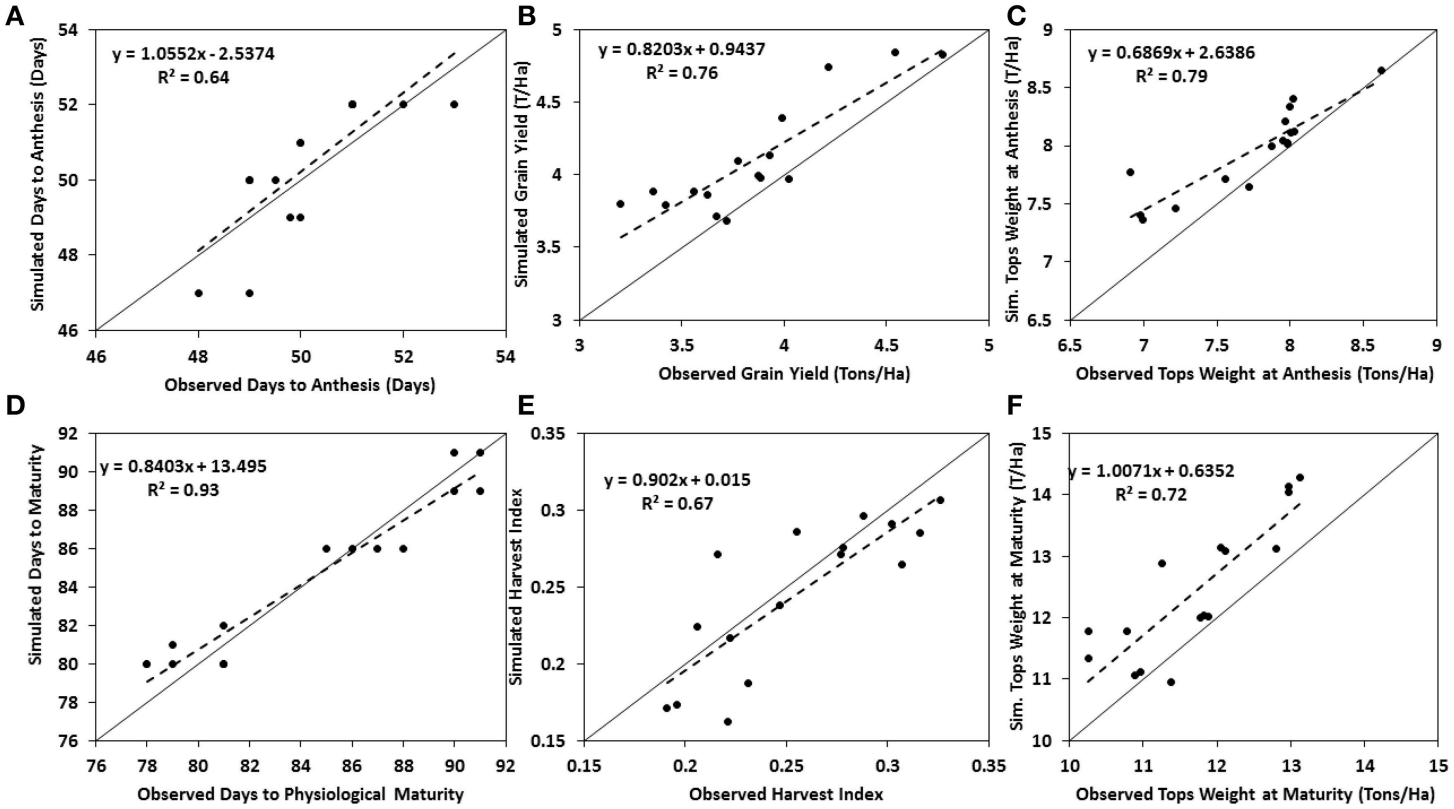
Comparisons of observed and simulated variables for the calibration of TZEE. Solid lines represent 1:1 relationships, dashed line represents linear regression, and each point represents a plot for both dry and rainy seasons. **(A)** Days to Anthesis; **(B)** Grain Yield, **(C)** Tops Weight at Anthesis, **(D)** Days to Physiological maturity, **(E)** Harvest Index, and **(F)** Tops Weight at Maturity.

**Figure 4:**
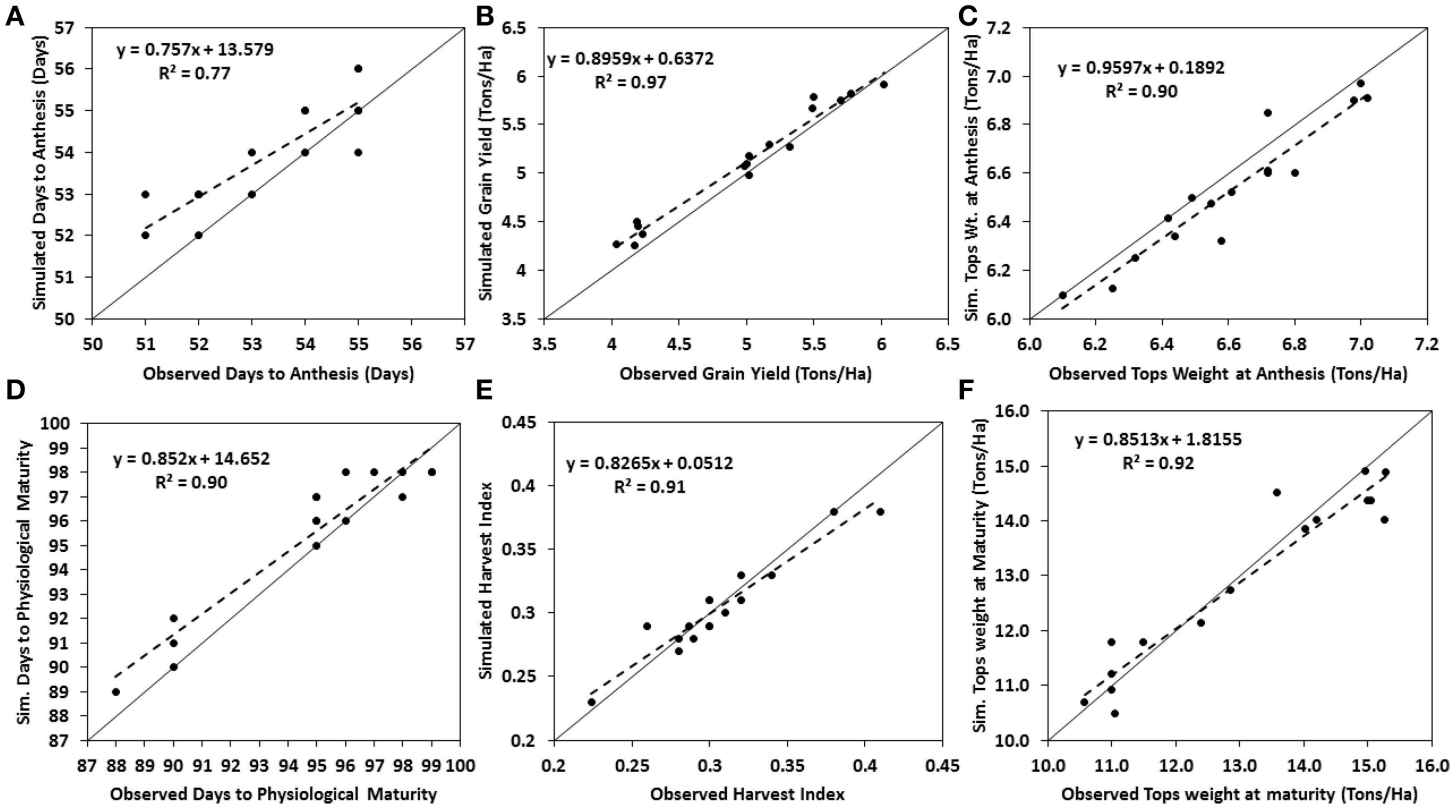
Comparisons of observed and simulated variables for the calibration of EVDT. Solid lines represent 1:1 relationships, dashed line represents linear regression, and each point represents a plot for both dry and rainy seasons. **(A)** Days to Anthesis; **(B)** Grain Yield, **(C)** Tops Weight at Anthesis, **(D)** Days to Physiological maturity, **(E)** Harvest Index, and **(F)** Tops Weight at Maturity.

Genotype specific parameters generated from the calibration experiments of the two varieties are presented in Table 2. Thermal time from seedling emergence to the end of juvenile phase (P1) for EVDT was 205 while that of TZEE was 196.1. Calculated value for P2 (Delay in development for each hour that day-length is above 12.5 h) was set as 0.5 for both varieties since both varieties are photo-insensitive. Yield determining parameters (P5, G2, and G3) were also higher for EVDT than TZEE, this makes EVDT to potentially have higher yield and longer maturity period than TZEE. After generating the coefficients, the model was evaluated for its ability to simulate days to anthesis, days to physiological maturity, tops weight at anthesis, tops weight at harvest, and grain yield at harvest maturity of the two varieties. This was done by comparing model simulated variables to actual observed variables from the field experiments and then calculating evaluation statistics. The model slightly over predicted all the parameters for both varieties, although it was within acceptable range. Tops weight at anthesis and at harvest were under-predicted for EVDT (Table 3). The model over predicted grain yield at harvest maturity by 212 Kg ha^−1^. The over prediction for days to anthesis and physiological maturity were not up to a full day. The mean observed grain yield for TZEE under rain fed and irrigated conditions were 3,883 and 4,018 Kg ha^−1^, respectively with lower RMSE observed under rain fed than irrigated (Table 4). For harvest index, a lower RMSE was observed under irrigated than rain fed. The mean grain yield of EVDT under rain fed and irrigated conditions were 4,989 and 5,216 Kg ha^−1^, respectively. Similarly, lower RMSE and higher *R*^2^-values were recorded for rain fed conditions than irrigated conditions for grain yield, days to anthesis, and days to maturity. Table 5 shows the mean and range for normalized root mean square error (RMSEn) and d index for model evaluation with time series data for maize grown during the 2014 and 2015 Seasons. The mean d index observed for TZEE and EVDT were 0.88 and 0.86, respectively, with a lower RMSEn recorded for TZEE. Figures 3, 4 show 1:1 lines between simulated and observed calibration parameters. For both varieties, better fits were observed for phenological variables when compared with yield and yield attributes. Generally, lower values of RMSEn were recorded for TZEE than EVDT but the ranges were wider for EVDT than TZEE. For stem and leaf dry weight, the d index and RMSEn-values were higher for EVDT than TZEE. Generally, phenological and yield parameters were simulated with higher accuracy than growth and biomass.

**Table 2:** Genotype specific parameters for maize varieties used.

**Coefficient**	**Description**	**EVDT**	**TZEE**
P1 (° days)	Thermal time from seedling emergence to the end of juvenile phase	205.0	196.1
P2 (days)	Delay in development for each hour that day-length is above 12.5 h	0.50	0.50
P5 (° days)	Thermal time from silking to time of physiological maturity	860	822
G2 (#)	Maximum kernel number per plant	652	630
G3 (mg day^−1^)	Kernel growth rate during linear grain filling stage under optimum conditions	7.8	6.6
PHINT (°C day tip^−1^)	Thermal time between successive leaf tip appearance	40.0	37.6

**Table 3:** Comparisons of predicted (Prd.) and observed (Obs.) mean days to anthesis, days to physiological maturity, tops weight at anthesis (Kg ha^−1^), tops weight at maturity (Kg ha^−1^), and grain yield at harvest (Kg ha^−1^) of early and extra early Maize^†^.

**Cultivars**	**Days to anthesis (#)**			**Days to Physiological maturity (#)**			**Tops weight at anthesis (Kg ha^−1^)**			**Tops weight at harvest (Kg ha^−1^)**			**Grain yield at harvest maturity (Kg ha^−1^)**		
	**Prd**.	**Obs**.	**PD^¶^**	**Prd**.	**Obs**.	**PD**	**Prd**.	**Obs**.	**PD**	**Prd**.	**Obs**.	**PD**	**Prd**.	**Obs**.	**PD**
TZEE	50	50.6	0.6	80	80.2	0.2	7,954	7,738	216	12,426	11,707	719	4,095	3,883	212
EVDT	54	53.3	0.7	95	94.7	0.3	6,530	6,607	−77	12,919	13,043	−124	5,106	4,989	118

¶ *PD, Prediction Deviation*.

† *Negative deviations indicate under-prediction while positive deviations indicate over-prediction*.

**Table 4:** Mean observed values for harvest index, days to anthesis, and grain yield per hectare of maize with corresponding RMSE, r-square, and EF-values combined for 2014 and 2015 under irrigated and rainfed conditions.

**Variable**	**TZEE**			**EVDT**		
	**Mean Obs.^a^**	**RMSE**	**r-square**	**Mean Obs**.	**RMSE**	**r-square**
**RAINFED TRIALS**						
Harvest index	0.25	0.04	0.63	0.31	0.04	0.91
Days to anthesis	50.5	2.37	0.70	53.3	1.96	0.91
Days to maturity	84.1	3.12	0.67	95.8	3.02	0.92
Grain yield ha^−1^	3,883	43.04	0.83	4,989	41.92	0.97
**IRRIGATED TRIAL**						
Harvest index	0.28	0.02	0.71	0.38	0.02	0.91
Days to anthesis	51.7	2.08	0.79	55.5	2.19	0.87
Days to maturity	85.7	3.25	0.77	97.1	4.77	0.85
Grain yield ha^−1^	4,018	86.34	0.89	5,216	107.86	0.88

**Table 5:** Mean and range for normalized root mean square error (RMSEn) and *d* index for model evaluation with time series data for maize grown during the 2014 and 2015 rainy and dry seasons.

**Crop character**	**RMSEn (%)**		***d*** **index**	
	**Mean**	**Range**	**Mean**	**Range**
**TZEE**				
Tops weight (ton/ha)	51	49–53	0.88	0.79–0.91
Stem dry weight	62	60–66	0.79	0.68–0.88
Leaf dry weight	64	58–66	0.81	0.73–0.92
**EVDT**				
Tops weight (ton/ha)	58	53–62	0.86	0.77–0.92
Stem dry weight	65	61–70	0.90	0.84–0.96
Leaf dry weight	68	66–71	0.91	0.86–0.98

#### Model evaluation

Table 6 shows the result of model evaluation including evaluation statistics for the two varieties in both locations. There was a good fit in the model prediction for grain yield with D-index and EF-values of 0.93 and 0.94, respectively, for TZEE at all locations in the Sudan Savanna while in the Northern Guinea Savanna, lower values were recorded for the same variety (0.85 and 0.86, respectively). Grain yield of EVDT was also simulated with high accuracy in both environments, with both D-index and EF-values recorded above 0.8. Stalk weight at anthesis and tops weight at physiological maturity also showed good predicted vs. observed fits, with D-index and EF-values above 0.8 in all cases, except for EVDT in Bunkure where D-index value of 0.77 was recorded. For days to anthesis, D-index, and EF-values were observed to be above 0.86 in both environments, with the highest D-index value (0.96) recorded for EVDT in Zaria and highest EF-value (0.97) recorded for the same variety at the same location. The values of D-index and EF for all the measured variables showed that observed and simulated characters were in good agreement with each other, which means that the model is robust and accurate in measuring both phenology and yield/yield attributes.

**Table 6:** Model evaluation statistics for evaluation experiments in Sudan and Northern Guinea Savannah for EVDT and TZEE.

**ANTHESIS DAYS (Number)**									
**Location**	**TZEE**					**EVDT**			
	**SIM**	**OBS**	***d*-Index**	***EF***		**SIM**	**OBS**	***d*-Index**	***EF***
Sudan Savanna	47.3	48	0.87	0.89		53.5	55	0.88	0.91
Northern Guinea	48.2	49	0.94	0.95		54.2	55	0.90	0.96
**DAYS TO PHYSIOLOGICAL MATURITY (NUMBER)**									
	**TZEE**					**EVDT**			
	**SIM**	**OBS**	***d*****-Index**	***EF***		**SIM**	**OBS**	***d*****-Index**	***EF***
Sudan Savanna	90.5	89	0.93	0.94		99.3	98	0.94	0.94
Northern Guinea	92.3	91	0.96	0.96		99.5	100	0.96	0.97
**GRAIN YIELD (Kg/ha)**									
	**TZEE**					**EVDT**			
	**SIM**	**OBS**	***d*****-Index**	***EF***		**SIM**	**OBS**	***d*****-Index**	***EF***
Sudan Savanna	3,491	3,396	0.93	0.94		5,335	5,298	0.88	0.89
Northern Guinea	3,986	4,211	0.85	0.86		6,014	5,834	0.82	0.84
**STALK WEIGHT AT ANTHESIS (Kg/ha)**									
	**TZEE**					**EVDT**			
	**SIM**	**OBS**	***d*****-Index**	***EF***	**RMSE**	**SIM**	**OBS**	***d*****-Index**	***EF***
Sudan Savanna	8,001	7,993	0.79	0.82	0.87	8,959	8,873	0.88	0.90
Northern Guinea	8,393	8,145	0.78	0.81	0.85	9,927	9,816	0.86	0.87
**Tops WEIGHT AT MATURITY (Kg/ha)**									
	**TZEE**					**EVDT**			
	**SIM**	**OBS**	***d*****-Index**	***EF***		**SIM**	**OBS**	***d*****-Index**	***EF***
Sudan Savanna	12,379	11,999	0.86	0.88		13,271	12,998	0.77	0.81
Northern Guinea	13,296	13,775	0.85	0.87		14,012	13,975	0.82	0.84

### Sensitivity analysis (model application)

The mean, maximum, and minimum simulated grain yields from 26 years' seasonal analysis for the different planting windows is shown in Table 7. When TZEE was planted in Bunkure, the highest grain yield was produced in early June. When planting was delayed to early, mid and late July, grain yields still remained within 3 tons per hectare threshold with the lowest grain yield recorded in the late July planting window. For EVDT however, the highest grain yield was observed when planting was done in late June contrary to TZEE. Planting EVDT in July produced high grain yields (<5,200 Kg/ha). Planting both varieties in early and mid-May in Bunkure produced minimum yields of 0 Kg/ha while delaying planting to mid and late August produced minimum grain yields of 1,254 and 742 Kg/ha for TZEE and 1,906 and 1,410 Kg/ha for EVDT, respectively. In Zaria however, TZEE produced highest grain yield (4,217 Kg/ha) when planting was done in late July. Yields above 4 tons/ha were observed for all planting dates from mid-May to late July. Delaying planting date to early and mid-August led to significant decline in grain yield (2,881 and 2,557 Kg/ha, respectively). Planting EVDT in Zaria produced similar response to TZEE, with the highest simulated grain yield of 6,079 produced in late July. The lowest minimum grain yield (1,622 and 1,738 Kg/ha) for TZEE and EVDT were observed when planting was delayed to late August. The highest maximum yields for both varieties (5,050 for TZEE and 6,966 EVDT Kg/ha) were observed when planting was done in late July. Yield reduction of only 15% was observed when planting of TZEE was delayed from June to July in Bunkure, while delaying planting further to August resulted in yield decline of 64.5%. In Zaria however, the yield reduction between planting in July and August was 66.9%. For EVDT, delaying planting from July to August led to a yield decline of 74.4% in the Bunkure and 94.3% in Zaria.

**Table 7:** Result of 26 year seasonal analysis using different planting windows for early and extra-early maize in Bunkure and Zaria.

**Sowing window**	**Grain Yield (Kg ha**^**−1**^**) TZEE Bunkure**				**Grain Yield (Kg ha**^**−1**^**) TZEE Zaria**			
	**Mean**	**Max**.	**Min**.	**St. Dev**	**Mean**	**Max**.	**Min**.	**St. Dev**
Mid May	3,096	4,499	0	474.5	4,125	4,436	3,462	253.8
Late May	3,390	4,261	0	674.6	4,123	4,609	1,901	556.4
Early June	3,887	4,613	2,243	486.2	4,090	4,525	2,676	393.1
Mid-June	3,837	4,632	2,596	260.7	4,163	4,586	3,564	284.1
Late June	3,522	4,625	2,391	442.1	4,169	4,606	3,202	382.4
Early July	3,318	4,575	2,402	406.6	4,175	4,750	2,937	444.6
Mid July	3,211	4,242	1,875	643.2	4,217	4,752	2,910	429.1
Late July	3,067	4,079	1,832	424.2	4,141	5,050	2,982	472.5
Early August	2,823	3,990	1,254	482.5	2,881	4,887	2,872	531.6
Mid-August	2,362	3,856	742	556.4	2,527	4,628	1,622	701.4
	**Grain Yield (Kg ha^−1^) EVDT Bunkure**				**Grain Yield (Kg ha^−1^) EVDT Zaria**			
Mid May	3,167	6,815	0	694.1	5,534	6,254	4,084	640.3
Late May	4,473	7,194	0	525.1	5,545	6,294	2,526	904.1
Early June	4,943	7,426	2,611	611.8	5,600	6,348	2,004	915.6
Mid-June	5,435	7,665	2,364	346.5	5,572	6,043	4,222	414.5
Late June	6,092	7,685	4,760	410.7	5,701	6,329	4,522	379.7
Early July	5,865	7,665	3,780	584.3	5,799	6,400	4,670	315.1
Mid July	5,846	7,665	3,601	359.2	5,939	6,400	5,091	414.2
Late July	5,281	7,124	3,560	733.1	6,079	6,966	5,116	646.3
Early August	3,571	5,898	1,906	815.2	3,782	6,536	3,758	640.2
Mid-August	3,493	5,195	1,410	694.1	3,128	6,889	1,785	644.3

**Figure 5:**
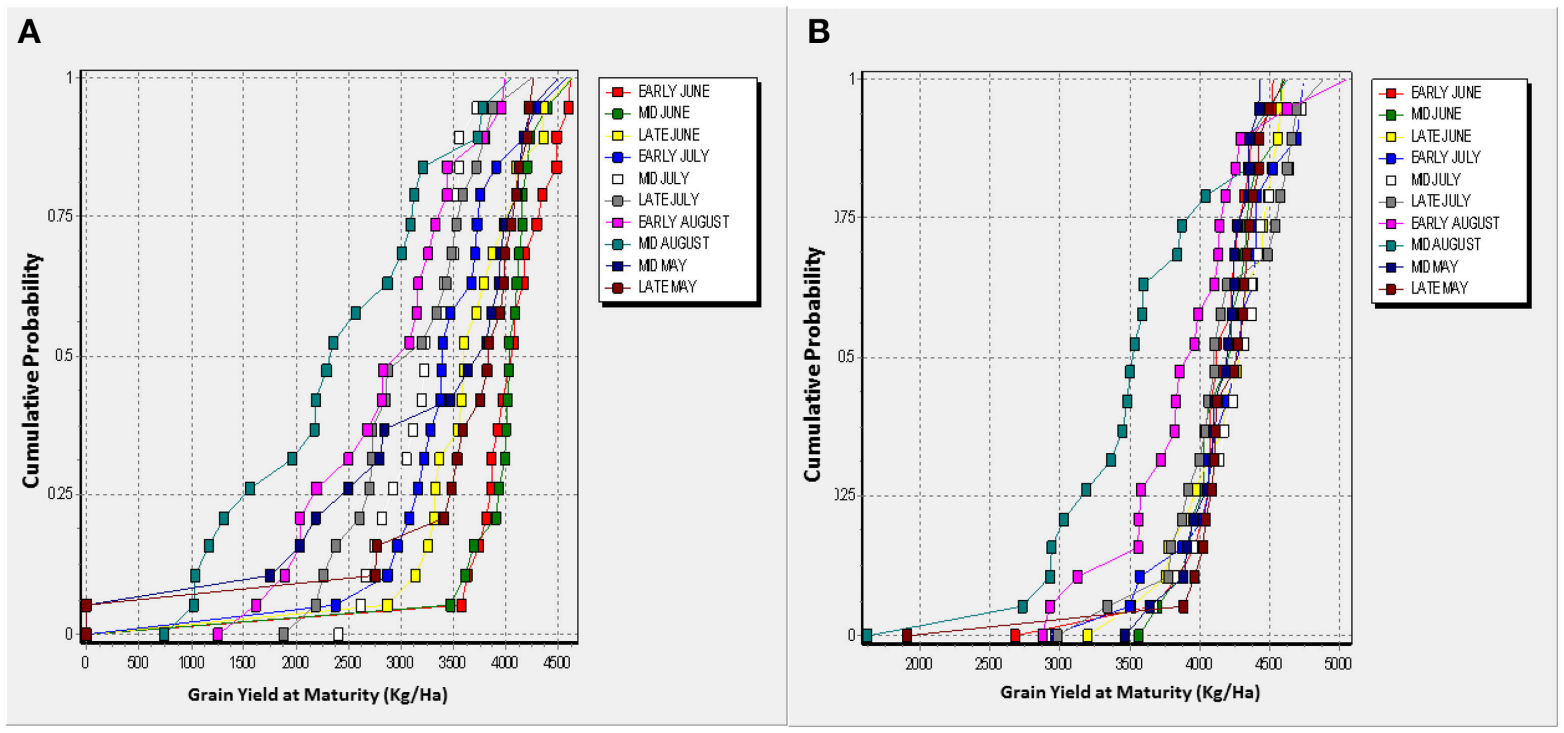
Cumulative function plots for simulated grain yields of TZEE in Bunkure **(A)** and Zaria **(B)**.

**Figure 6:**
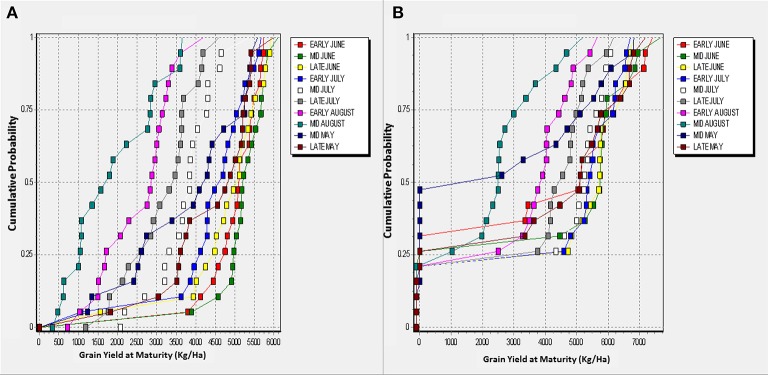
Cumulative function plots for simulated grain yields of EVDT in Bunkure **(A)** and Zaria **(B)**.

Figures 5A,B, 6A,B shows cumulative function plots for simulated grain yields of TZEE and EVDT in Bunkure and Zaria. The CF plots shows that for TZEE, delaying planting to August produced yields below 3,000 Kg/ha more than 75% of the time in Bunkure. For EVDT however, yields below 4 tons were observed when planting was done in August with probability of 0.5. Planting TZEE in early June and EVDT in late July produced the highest grain yield more than 90% of the time in Bunkure. In Zaria however, planting in early and mid-August leads to low yields with more than 80% probability. The probability of getting high yields for TZEE was highest (0.8) when planting was done in late July. Highest grain yields were observed for EVDT when planted in early June (probability = 0.75). Planting TZEE in mid and late May at Bunkure produced 0 yields with probability of 0.1. The tendency of having 0 yields as a result of planting in May was higher (0.4) when EVDT was planted in mid and late May at Zaria.

## Discussions

Sowing date recommendations for maize in Nigeria are usually based on local knowledge. Recommendations are made from large-scale cropping experiments conducted across regions (NAERLS, 2013). Most of the time, the same sowing date is recommended for multiple years and multiple locations without considering seasonal and spatial variations. Farmers also take risk by planting with the first onset of rain because of the uncertainty of rainfall duration in the Nigerian savannas. Wolf et al. (2015) suggested that sowing rules in Sub-Saharan Africa should have a time window that is at most 40 days around the roughly estimated best date of sowing. The recommended sowing date for maize in Nigeria is early to mid-June in both SS and NGS (NAERLS, 2013). Findings from our research shows that variations exist between varieties and locations with respect to best sowing dates. The locations are influenced by agro-ecological zones.

The close agreement between observed and simulated variables for both calibration and evaluation experiments means that the model can be used to predict performance of the two varieties across different environments in Savannas of Nigeria. The outcomes of simulations resulted in high D-index, RMSE, and EF-values across all treatments and locations and for all tested variables indicating that the efficiency and robustness of the model is quite adequate and the model can be used in the environments under study. In CERES–maize model, flowering and maturity dates were controlled by the coefficients P1 and P5 in the genotype file. Accurate prediction of phenology was observed due to the close agreements between observed and simulated days to tasseling and days to physiological maturity for the calibration experiments. Accurate prediction of maize phenology is the most important stage in model calibration (Archontoulis et al., 2014). When phenology is accurately calibrated, it is expected that models will be able to capture all genotypic variations that affect the leaf area development, biomass production, and grain yield (Robertson et al., 2002). Grain yield is affected by radiation interception by crop canopy, radiation use efficiency (RUE) and harvest index (Lee and Tollenaar, 2007). Pantazi et al. (2016) suggested that yield prediction in crop modeling is the most important variable for the improvement of crop management. The close agreement between observed and simulated grain yield in both calibration and evaluation experiments can be attributed to accurate measurement of G2 and G3 and also to adjustments made to SLPF and RUE in the cultivar files of CERES–maize model. The high agreement between observed and simulated values for the evaluation experiments shows that the model is robust and accurate enough to make wider applications across the ecology under study. The result of evaluation trials using different planting dates indicates that the extra early maize varieties produced higher yields when planted in early June in the SS and in mid and late July in the NGS.

The result of both seasonal and sensitivity analysis indicates that the variation in yield for the different sowing dates tested was very high. When earlier (Mid and Late May) and later (early and mid-August) dates were simulated, higher variations in yield were observed. This is an indication that early planting, which is a norm by farmers in the Nigerian Savannas is not only risky, but it could lead to high reduction in yield of maize. Also, early planting at the onset of rainy season is quite risky, as most of the time early rains are followed by long dry periods which could lead to total crop failure. Late planting also leads to a higher yield reduction and has the potential of resulting in total crop failure. Late planting results in yield reduction due to failure of the crops to mature if the rainfall ceases early before the end of the cropping season. This will also have a detrimental effect on the final grain yield (Lauer, 1998; Jibrin et al., 2012). In the Nigerian Savannas, the rainy season establishes in late June and ends in October. Late plating in August will not allow the plants to complete their life cycle before the end of the rainy season. Maize crops planted in August will therefore experience severe drought stress at flowering stage which is critical for maize productivity. The high yields observed for planting in mid and late July means that maize farmers in the Sudan and Northern Guinea Savannas can get reasonably high yields in seasons where delay in rainfall establishment is experienced. For the early varieties, higher yields were observed when planted in late June in the SS and mid-late July in the NGS. This is a clear indication that early and extra-early varieties could be planted in places where delay in onset of rainy season is experienced. The delay in establishment of rainfall is becoming prevalent in the Nigerian savannas, thus the result of the seasonal analysis means that planting early and extra early maize is best delayed until the first or second week of July in both savannas. The result of this work is in agreement with findings by Kamara et al. (2009) who reported higher grain yield of maize in the Sudan Savanna, although on different soils, when planting was delayed to early and mid-July. Also Jibrin et al. (2012) reported that CERES–maize model predicted decrease in grain yield with delay in planting date to early August except for TZB-SR at Azir, North-East Nigeria in 2006 where planting on July 13 gave higher yield than planting on June 29. The reason for differential response to maize planting dates could be attributed to variation in the maturity periods. Extra early and early varieties complete their life cycles earlier as a result there is room to delay planting especially in the Northern Guinea Savanna. Jaliya et al. (2008) made similar findings from field trials with different maturing maize varieties, they reported that planting in mid-June to late July in both Sudan and Northern Guinea Savannas leads to high yields of maize. They also reported that when early planting is done before proper establishment of rains low population as well as poor plant vigor/establishment could be experienced this might lead to reduction in yield. Late planting results in flowering coinciding with cessation of rains, this could lead to reduction in number of kernels/cob and drastic reduction in final yield.

## Conclusion

The ability of CERES–maize to reasonably predict phenology, grain yield and tops weight of the varieties used in this study is an indication of its usefulness as a decision-support tool for maize researchers and extension workers in the Savanna regions of Nigeria. The Model suggests that both early and extra-early varieties yield higher when planted in mid to late June in SS, and mid-late July in NGS. While both varieties yield higher when planted in mid to late July in the NGS. Delays in planting to August can result in significant yield reductions. In both SS and NGS planting in May and August are quite risky and could lead to total crop failure.

## Author contributions

AA conducted most of the field experiments as well as simulations in this publication as part of his Master and Doctorate research. He was responsible for most of the manuscript preparation and is the lead author. JJ is AA's major supervisor and he helped in providing major insight into both experimental and simulated outputs. He helped in manuscript preparation and provided major input into soil and weather information. AK is also a member of the supervisory team. He gave major inputs into understanding the outcomes of experimental and modeled output. He also helped in manuscript preparation and gave deeper understanding of sensitivity analysis output. BA was involved in field management, data collection, soil analysis, and weather data generation and input. He was responsible for most of the soil and weather information used in the research. AS helped in field management, data analysis, and graphics. He also helped in running the tools responsible for generation of genetic coefficients used in the research. IG was also involved in field data collection and generation of simulation runs. He also helped in manuscript preparation and generation of graphs.

### Conflict of interest statement

The authors declare that the research was conducted in the absence of any commercial or financial relationships that could be construed as a potential conflict of interest.
